# Mammographic density and breast cancer risk: the role of the fat surrounding the fibroglandular tissue

**DOI:** 10.1186/bcr3044

**Published:** 2011-12-03

**Authors:** Mariëtte Lokate, Petra HM Peeters, Linda M Peelen, Gerco Haars, Wouter B Veldhuis, Carla H van Gils

**Affiliations:** 1 Julius Center for Health Sciences and Primary Care, Str. 6.131, University Medical Centre Utrecht, PO Box 85500, 3508 GA Utrecht, The Netherlands; 2 Julius Clinical Research, J.F. Kennedylaan 101 III,3981 GB Bunnik, The Netherlands; 3 Department of Radiology, E01.132, University Medical Centre Utrecht, PO Box 85500, 3508 GA Utrecht, The Netherlands; 4 Department of Epidemiology and Biostatistics, School of Public Health, Faculty of Medicine, Imperial College London, St. Mary's Campus, Norfolk Place W2 1PG London, UK

## Abstract

**Introduction:**

Both the percent of mammographic density and absolute dense (fibroglandular) area are strong breast cancer risk factors. The role of non-dense (fat) breast tissue is not often investigated, but we hypothesize that this also influences risk. In this study we investigated the independent effects of dense and fat tissue, as well as their combined effect on postmenopausal breast cancer risk.

**Methods:**

We performed a nested case-control study within the EPIC-NL cohort (358 postmenopausal breast cancer cases and 859 postmenopausal controls). We used multivariate logistic regression analyses to estimate breast cancer odds ratios adjusted for body mass index and other breast cancer risk factors.

**Results:**

Large areas of dense (upper (Q5) vs lower quintile (Q1): OR 2.8 95% CI 1.7 to 4.8) and fat tissue (Q5 vs Q1: OR 2.4; 95% CI 1.3 to 4.2) were independently associated with higher breast cancer risk. The combined measure showed that the highest risk was found in women with both a large (above median) area of dense and fat tissue.

**Conclusions:**

Fibroglandular and breast fat tissue have independent effects on breast cancer risk. The results indicate that the non-dense tissue, which represents the local breast fat, increases risk, even independent of body mass index (BMI). When studying dense breast tissue in relation to breast cancer risk, adjustment for non-dense tissue seems to change risk estimates to a larger extent than adjustment for BMI. This indicates that adjustment for non-dense tissue should be considered when studying associations between dense areas and breast cancer risk.

## Introduction

In the last two decades, many researchers have observed a strongly elevated breast cancer risk in women with a high percent mammographic density [1,2]. Percent mammographic density represents the relative amount of fibroglandular tissue, which is radiographically dense, and fat tissue, which is radiographically lucent. A high percent mammographic density is associated with a three-to-six-fold increase in breast cancer risk comparing the extremes of the breast density distribution [2].

Increasingly, the absolute area of dense tissue is reported in the literature along with the percent density measure. The reason for this is that the dense area is considered to represent the actual target tissue for tumor development [3,4]. As percent density is strongly influenced by the size of the fat area, or non-dense tissue, in the breast using a percentage seems less appropriate. Several studies show approximately equal results for percent density and absolute dense area [1,5-7], although some others show stronger [8] or weaker results for the absolute dense area [9-11].

Until now, there has been little attention given to the role of non-dense breast tissue, despite the fact that fat cells are known to be highly active endocrine cells that secrete numerous hormones which are thought to be important for the development of breast cancer [12,13]. The interaction between body mass index (BMI) and breast density in relation to breast cancer has been investigated a few times [11,14-17], but only two studies investigated the role of the non-dense breast tissue in relation to breast cancer [18,19]. Stuedal *et al. *studied this indirectly, by assessing whether a large area of dense tissue is more harmful in small or large breasts, or in other words, in combination with a small or large area of fat tissue. In a combined population of African American and white women, Stuedal *et al. *observed that the association between percent and absolute mammographic density and breast cancer risk was weaker in women with larger breasts [18]. One of their explanations was that fat tissue has a potential protective effect on breast cancer [18]. Although experimental literature provides some evidence for this [20], most experimental studies suggested that fat tissue secretes proteins which could stimulate proliferation of malignant cells [21-23]. It is also known that the fat tissue produces estrogens by the conversion of androgens and increases breast cancer risk [24-27].

Stone *et al. *also studied which breast tissue characteristics best predict breast cancer risk: dense area, non-dense area, percent density or a combination thereof. They observed that the dense area was a better predictor than percent density. Further addition of non-dense area did not alter the fit of the model, suggesting no additional role for the fat tissue in influencing breast cancer. Unfortunately, no information on breast cancer risk factors, other than age, was available for inclusion in their models [19].

To obtain more insight into the role of the fat breast tissue, we investigated the independent effects of the size of dense tissue and the size of non-dense tissue in the breast and their combined effects on breast cancer risk in a nested case control study of postmenopausal Caucasian women.

## Methods

### Study population

The study population comprises participants of the Prospect-EPIC study [28], which is a part of the EPIC-NL study [29]. EPIC-NL is the Dutch contribution to the European Prospective Investigation into Cancer and Nutrition (EPIC) study [30,31]. The Prospect-EPIC participants were recruited through the breast cancer screening programme in Utrecht and vicinity. These included 17,000 women aged between 49 and 70 years at recruitment [28]. At the time of recruitment, anthropometric parameters were measured and the participants filled out extensive questionnaires yielding information on demographic, lifestyle and reproductive factors, and past and current morbidity.

Incident breast cancer cases were identified by linking the cohort data with the regional cancer registry, IKMN (Integraal Kankercentrum Midden Nederland), and the national cancer registry, LKR (Landelijke Kanker Registratie). Until 2007, 607 breast cancer cases had been identified among the 17,000 breast cancer screening participants. For each breast cancer case, the researchers used incidence density sampling to select three controls from the cohort [32]. In total, 1,821 controls were selected. Breast cancer cases and controls were excluded if they previously had a diagnosis of any type of cancer, except non-melanoma skin cancer, or if they had less than three mammograms, which was a prerequisite for a study on changes in mammographic density [publication in preparation]. If a person was excluded, the respective controls were excluded too. In total, 475 breast cancer cases and 1,187 controls were eligible for this study. As different aetiology for breast cancer in postmenopausal women (1,217 women) compared with premenopausal women (370 women) is likely, especially for the role of body fat, we only selected the postmenopausal women. In addition, 22 breast cancer cases and 52 controls were excluded because they had breast implants or the mammogram could not be retrieved, resulting in a final study population of 358 cases and 859 controls.

All participants gave their informed consent and the study was approved by The Institutional Review Board of the University Medical Centre Utrecht.

### Mammographic density assessment

Mammographic density was assessed on the left mediolateral oblique mammograms (MLO) taken at the time of recruitment into the cohort, on average six years before diagnosis. The mammograms (all film screen) were digitized with a Canon CFS300 scanner (R2 Technology, Grand Rapids, MI, USA) with a pixel resolution of 50 *μ*m and 12 bits per pixel.

For assessing the mammographic density, Cumulus software (University of Toronto, ON, Canada) was used [33]. With this software, two thresholds were set by the reader; one to distinguish the breast area from the background and a second to distinguish the dense from the non-dense area. Furthermore, the pectoralis muscle was masked out. The non-dense area was obtained by subtracting the dense area from the total breast size. The size of the dense and non-dense areas in cm^2^, was determined by multiplying the number of pixels in the respective areas by the size of one pixel. Percent density was calculated by dividing the dense area by the total breast area and multiplying by 100. All mammograms were read by one single reader (ML), blinded to participant characteristics, in batches of 53 mammograms each. Mammograms of cases and corresponding controls were in the same batch but randomly ordered. Each batch included two or three duplicate mammograms to allow estimation of the within-batch intraclass correlation coefficient. Also, a test batch was read before, after and three times in between the batches to determine the between-batch intraclass correlation coefficient. The intraclass correlation coefficients for the within-batch correlation as well as the between-batch correlation were 0.96, 0.96 and 1.00 for percent density, dense area and breast area, respectively.

### Statistical analysis

Patient characteristics were described within quintiles of dense area and non-dense area among controls. For continuous variables we used means with standard deviations if normally distributed and medians with interquartile ranges of not normally distributed data (number of children). For categorical variables, we show proportions. To examine the presence of a linear trend in the distribution of the risk factors over the dense area and non-dense area quintiles, we used a test for linearity for normally distributed continuous data, a Jonckheere Terpstra test for skewed continuous data (number of children) and a chi-square linear trend test for proportions.

Correlations between density measures with BMI and each other are calculated using the Pearson correlation coefficient. The non-linear density measures were transformed using natural logarithm in order to get normally distributed measures.

We examined the relationship between breast measures and breast cancer risk by calculating odds ratios and their 95% confidence intervals (95% CI) for quintiles of percent density, dense area and non-dense area. Quintiles were based on the distributions of controls. Multivariate logistic regression was used to adjust for age at mammography, age at menarche, age at first delivery (nulliparous, ≤25 years at first delivery, >25 years at first delivery), number of children, age at menopause, hormone therapy (HT) use (never, past, current), birth control pill use (never, past, current), first degree relative with a history of breast cancer, that is, mother or sister (no/yes), height and BMI. To investigate the independent effects of the dense and the non-dense areas, the analyses of the dense area were additionally adjusted for the non-dense area. Despite the strong correlation between BMI and the non-dense area, we included the non-dense area in addition to BMI to examine whether the local breast fat has an effect on breast cancer risk independent of BMI. The analyses of the non-dense area were additionally adjusted for the dense area. The analyses are also additionally adjusted for BMI.

In order to investigate the joint effect of dense and non-dense tissue, these variables were dichotomized by the median value of their distributions in the controls. Four groups were defined, that is, women with small areas of dense and non-dense tissue (low/low, reference category), women with a small area of dense but a large area of non-dense tissue (low/high), women with a large area of dense, but a small area of non-dense tissue (high/low) and those with a large area of both dense and non-dense tissue (high/high). Odds ratios were adjusted for the same confounders as described above. The relative excess risk (RERI) of women with above median areas of dense and non-dense tissue was calculated to assess the level of additive interaction.

All *P*-values are two-sided and if below 0.05 the results were considered statistically significant. Analyses were conducted using PASW Statistics 17.0 (SPSS, Inc., Chicago, IL, USA).

## Results

At recruitment, breast cancer cases were 60 years (SD 5.4) and controls 59 years (SD 5.6) on average. The breast cancer cases were older at the birth of their first child than the controls (26 (SD 4.2) vs 25 (SD 4.0) years) and also older when they became postmenopausal (48 (SD 5.6) vs 47 (SD 5.7) years). The mean age at diagnosis of the breast cancer cases was 66 years (SD 5.8) and the median time between the recruitment and the diagnosis was 6 years (interquartile range: 4 to 9 years).

Distributions of breast cancer risk factors among quintiles of dense tissue and non-dense tissue in controls are presented in Table 1. Women who were older or had a higher BMI had a smaller absolute area of dense tissue, whereas women who were nulliparous had larger areas of dense tissue (Table 1). Larger areas of non-dense tissue were observed in women who were older or had a higher BMI. Women had a smaller area of non-dense tissue when they were nulliparous.

**Table 1:** Breast cancer risk factors by density measurs (quintiles* in controls (N = 859)

	*Absolute dense area*						*Absolute non-dense area*					
	Q1	Q2	Q3	Q4	Q5		Q1	Q2	Q3	Q4	Q5	
	(172)	(172)	(171)	(172)	(172)		(172)	(171)	(172)	(173)	(171)	
	*Mean (sd)*					*P-trend*	*Mean (sd)*					*P-trend*
Age at examination (years)	60.3 (5.3)	60.6 (5.5)	59.1 (5.4)	58.8 (5.7)	56.7 (5.3)	**<0.001**	57.2 (5.1)	57.8 (5.3)	59.7 (5.4)	59.8 (5.5)	60.9 (5.6)	**<0.001**
BMI (kg/m^2^)	27.7 (4.1)	26.4 (4.0)	25.6 (3.5)	25.3 (3.7)	25.1 (3.9)	**<0.001**	22.9 (2.4)	24.4 (2.5)	25.9 (2.8)	26.9 (3.3)	30.0 (4.4)	**<0.001**
Height (cm)	163.4 (5.8)	163.8 (7.2)	164.1 (6.3)	164.4 (6.7)	165.0 (5.5)	**0.02**	164.7 (5.8)	164.3 (6.5)	163.6 (5.9)	164.0 (6.8)	164.0 (6.5)	0.20
Age at menarche(years)	13.4 (1.6)	13.5 (1.7)	13.3 (1.5)	13.5 (1.7)	13.3 (1.5)	0.72	13.6 (1.5)	13.4 (1.7)	13.4 (1.6)	13.2 (1.6)	13.3 (1.6)	**0.05**
Age at menopause (years)	47.1 (6.1)	47.9 (5.5)	47.1 (5.6)	48.0 (5.5)	46.9 (5.9)	0.87	47.8 (4.9)	47.4 (6.1)	47.2 (5.9)	47.1 (5.9)	47.5 (5.7)	0.45
Nr. of children^†^	2.9 (1.4)	2.6 (1.6)	2.5 (1.5)	2.3 (134)	2.1 (1.4)	**<0.001**	2.1 (1.3)	2.3 (1.5)	2.6 (1.4)	2.7 (1.6)	2.8 (1.5)	**<0.001**
Age at first delivery (years)^‡^	25.0 (4.0)	25.4 (4.5)	24.8 (3.8)	25.2 (3.7)	24.9 (4.1)	0.87	25.4 (3.6)	24.9 (3.9)	24.7 (3.8)	25.4 (4.5)	25.0 (4.3)	0.79
	***N (%)***						***N (%)***					

Nulliparous	9 (5.2)	16 (9.3)	20 (11.7)	23 (13.4)	33 (19.2)	**<0.001**	32 (18.6)	23 (13.5)	17 (9.9)	16 (9.2)	13 (7.6)	**0.001**
HT use (ever)	32 (18.6)	40 (23.3)	39 (22.8)	56 (32.7)	50 (29.1)	**<0.01**	50 (29.2)	48 (28.1)	41 (23.8)	39 (22.5)	39 (22.8)	0.08
Pill use (ever)	106 (61.6)	117 (68.4)	107 (62.6)	112 (65.1)	109 (63.4)	0.99	112 (65.1)	107 (62.6)	115 (66.9)	110 (63.6)	107 (62.9)	0.77
Family history of breast cancer	28 (16.7)	20 (12.0)	27 (16.0)	23 (14.0)	26 (15.6)	0.99	29 (17.4)	25 (14.8)	21 (12.4)	20 (12.0)	29 (17.7)	0.80

* Quintiles by controls only

^‡ ^Parous women only

^† ^The median nr. of children is shown in this table

Bold p-trends are statistically significant

The measurements of the different breast tissues showed that the breast cancer cases compared to the controls (unadjusted) had a larger median area of dense tissue (18.5 cm^2 ^(IQR 9.5 to 30.3) versus 14.9 cm^2 ^(IQR 6.9 to 29.7)) and non-dense tissue (119.3 cm^2 ^(IQR 87.5 to 163.5)) versus 116.4 cm^2 ^(IQR 84.7 to 147.5)). The median percent density also was higher in breast cancer cases than in controls (13.3% (6.4 to 23.4) versus 11.3% (4.7 to 23.8)) (not in Table 1).

In Table 2 it is shown that, as expected by the way it is calculated, a higher percent density is strongly positively correlated with a higher dense area (0.96, *P *< 0.001) and strongly negatively correlated with a higher non-dense area (-0.70, *P *< 0.001). The dense area itself, however, is also negatively correlated with the non-dense area (-0.47, *P *< 0.001). The non-dense area is strongly positively correlated with BMI (0.59, *P *< 0.001), and the dense area weakly negatively (-0.21, *P *< 0.001).

**Table 2:** Pearson Correlations of density measures with each other and with BMI*

	*Breast area*	*Dense area*	*Non-dense area*	*Percent density*	*BMI*
***Breast area***	*1*	*-0.18*	*0.90*	*-0.46*	*0.62*
***Dense area***		*1*	*-0.47*	*0.96 *	*-0.21 *
***Non-dense area***			*1*	*-0.70*	*0.59*
***Percent density***				*1*	*-0.37*
***BMI***					*1*

*The p-values of all correlations are statistically significant (< 0.001)

As shown in Table 3, a high percent density was associated with higher breast cancer risk (Q5 vs Q1 OR: 1.8, 95% CI: 1.0 to 2.9, *P *for trend: 0.002). The risk estimates for dense area seem to be somewhat stronger than those for percent density although both risk estimates are within the confidence intervals of each other (Q5 vs Q1 OR: 2.8, 95% CI: 1.7 to 4.8, *P *for trend: < 0.001).

**Table 3:** Breast tissue measures and breast cancer risk

*Quintiles*	*N (Case/Controle)*	*Median (%)* *(IQR)*	OR (95% CI)*	OR (95% CI)**	*OR* (95% CI)***	OR (95% CI)****
Percent Density						
1	53/171	2.2 (1.5 to 3.0)	**Ref**	**Ref**		
2	61/171	5.8 (4.8 to 7.0)	1.4 (0.9 to 2.3)	1.6 (1.0 to 2.5)		
3	88/172	11.4 (10.0 to 13.4)	1.8 (1.1 to 3.3)	2.1 (1.3 to 3.3)		
4	88/173	20.3 (18.1 to 23.5)	2.1 (1.6 to 3.4)	2.5 (1.6 to 4.1)		
5	68/172	38.9 (38.9 to 48.9)	1.4 (1.0 to 2.9)	1.8 (1.0 to 2.9)		
***P-trend***			*0.029*	*0.002*		
Dense Area^†^		**Median (cm^2^)** **(IQR)**				

**1**	46/172	3.6 (2.3 to 4.6)	**Ref**	**Ref**	**Ref**	**Ref**
**2**	55/172	8.3 (6.9 to 9.5)	1.3 (0.8 to 2.1)	1.4 (0.9 to 2.4)	1.5 (0.9 to 2.4)	1.5 (0.9 to 2.4)
**3**	86/171	14.9 (12.6 to 16.8)	2.1 (1.3 to 3.3)	2.3 (1.4 to 3.7)	2.6 (1.6 to 4.2)	2.6 (1.6 to 4.2)
**4**	93/172	25.5 (22.6 to 29.3)	2.4 (1.5 to 3.8)	2.6 (1.7 to 4.2)	3.2 (2.0 to 5.3)	3.2 (2.0 to 5.3)
**5**	78/172	44.4 (39.5 to 54.4)	1.9 (1.1 to 3.0)	2.1 (1.3 to 3.4)	2.9 (1.7 to 4.9)	2.8 (1.7 to 4.8)
***P-trend***			*0.001*	*<0.001*	*<0.001*	*<0.001*
Non Dense Area ^‡^		**Median (cm^2^)** **(IQR)**				

**1**	65/172	61.0 (48.2 to 69.9)	**Ref**	**Ref**	**Ref**	**Ref**
**2**	61/171	90.5 (84.2 to 96.5)	1.0 (0.6 to 1.5)	0.9 (0.6 to 1.5)	1.1(0.7 to 1.8)	1.1 (0.7 to 1.8)
**3**	72/172	115.5 (109.4 to 121.3)	1.2 (0.8 to 1.9)	1.2 (0.7 to 1.8)	1.6 (1.0 to 2.5)	1.5 (0.9 to 2.4)
**4**	65/173	141.7 (133.2 to 148.9)	1.0 (0.6 to 1.6)	0.9 (0.6 to 1.5)	1.4 (0.9 to 2.3)	1.4 (0.8 to 2.3)
**5**	95/171	186.7 (172.1 to 211.8)	1.7 (1.1 to 2.5)	1.4 (0.9 to 2.4)	2.6 (1.6 to 4.2)	2.4 (1.3 to 4.2)
***P-trend***			*0.009*	*0.115*	*<0.001*	*<0.001*

* Potential confounders

** Potential confounders and BMI

*** Potential confounders and Breast Tissue (^† ^Adjusted for Non-Dense Area, ^‡ ^Adjusted for Dense Area)

**** Potential confounders, BMI and Breast Tissue (^† ^Adjusted for Non-Dense Area, ^‡ ^Adjusted for Dense Area)

Potential confounders: Age at mammography, Height, Age at Menarche, Age First Delivery (Nulliparous, ≤25 years at first delivery, >25 years at first delivery), Nr. of children, Age Menopause (Premenopausal, ≤50 years at menopause, >50 years at menopause), HT use (Never, Current, Ever), Pill use (Never, Current, Ever), Family History (No/Yes).

Comparing the analysis without adjusting for non-dense area and with adjusting for non-dense area, it can be observed that adding the non-dense breast area somewhat increases risk estimates even after BMI has already been included in the model, although the confidence intervals partly overlap (fourth vs second 'OR' column of Table 3). When BMI was excluded from the full model that also includes the non-dense area, risk estimates remained essentially the same (third vs fourth 'OR' column of Table 3). When dense area, non-dense area and BMI were all included in the same model, only the effect estimates of the dense and non-dense tissue area remained statistically significant.

After adjustment for dense area, a large non-dense area was also related with a higher breast cancer risk (Q5 vs Q1 OR: 2.4 95% CI 1.3 to 4.2, *P *for trend: < 0.001).

Again, exclusion of BMI did not further change the risk estimates (third vs fourth 'OR' column of Table 3).

Since BMI and non-dense area are closely related (correlation coefficient: 0.59) multicollinearity may have affected these models. To circumvent this problem we repeated analyses, including BMI and the residuals of non-dense area regressed on BMI. This did, however, not lead to different results.

Women with a large (above median, that is, >14.9 cm^2^) area of dense tissue and a small (below median, that is, <116.4 cm^2^) area of non-dense tissue showed a slightly higher breast cancer risk than with a small area of both tissue types (OR 1.6, 95% CI 1.0 to 2.5). The highest risk was observed in women with large areas of both dense and non-dense tissue (OR 1.8, 95% CI 1.1 to 3.0). This risk for women with high dense and non-dense area combined was slightly higher than the sum of the risk for women with large areas of either one of the high risk tissues, but not statistically significantly (RERI 0.24, 95% CI: -0.5 to 1.0). Women with a larger breast size do not necessarily have a higher breast cancer risk, as can be observed from the median breast sizes for the risk categories in Table 4. Women within the 'high dense - low non-dense' category have a significantly increased risk, whereas women in the 'low dense - high non - dense' category do not show an increased risk, despite both categories have larger breasts.

**Table 4:** Combined effect of dense and fat tissue on breast cancer risk

	*N Case/Control*	*Dense area* *(min.-max.)*	*Non-dense area* *(min.-max.)*	*Median breast size*	*Median percent density*	OR*; 95% CI	OR**; 95% CI
Low Dense^† ^- Low Non-Dense^‡^	41/138	0.8-14.9 cm^2^	38.3-116.3 cm^2^	102.9 cm^2^	9.34%	**Ref.**	**Ref.**
Low Dense - High Non-Dense	102/291	0.3-14.7 cm^2^	116.4-289.6 cm^2^	164.6 cm^2^	3.54%	1.18; 0.78-1.85	1.02; 0.64-1.63
High Dense - Low Non-Dense	129/289	15.1-98.2 cm^2^	12.5-116.1 cm^2^	115.3 cm^2^	28.24%	1.58; 1.02-2.44	1.58; 1.02-2.45
High Dense - High Non-Dense	86/139	14.9-99.2 cm^2^	116.6-258.6 cm^2^	175.1 cm^2^	13.69%	2.15; 1.34-3.45	1.84; 1.12-3.02

**RERI**						*0.39; -0.43-1.20*	*0.24; -0.52-1.00*

*Adjusted for age at examination, height, age at menarche, age at first delivery (nulliparous, ≤25 years at first delivery, >25 at first delivery), number of children, age at menopause (premenopausal, ≤50 years at menopause, >50 years at menopause), HT use (never, current, ever), pill use (never, current, ever), family history of breast cancer (yes/no)

**Same as *, but additionally adjusted for BMI

^†^Dense area was split by the median which was 14.9 cm^2^

^‡^Non-dense area was split by the median which was 116.4 cm^2^

## Discussion

In this paper, we investigated the role of dense and fatty breast tissue in relation to breast cancer risk. We found that not only a large area of fibroglandular tissue is associated with a higher breast cancer risk, but also that a large area of fat breast tissue, even after taking BMI into account, has an independent effect on breast cancer risk. Women who have both a large area of fibroglandular tissue and a large area of fat tissue seem to have the highest breast cancer risk. It should be noted that the relationship with breast cancer is stronger for the dense than for the non-dense area. Therefore, the resulting risk depends on the composition of the breast and not simply on the size of the breast. When studying dense breast tissue in relation to breast cancer risk, adjustment for non-dense tissue seems to change risk estimates to a larger extent than adjustment for BMI. This indicates that adjustment for non-dense tissue should be considered when studying associations between dense area and breast cancer risk.

The harmful effect of a large area of dense tissue on breast cancer risk is established, and could be explained by the fact that proliferating cells are the actual target tissue for breast cancer development [3,34]. Also, it has often been hypothesized that the area of the dense tissue would reflect the effect of estrogens on the breast, because many determinants of mammographic density are related to hormones (parity, menopause, hormone therapy) [34]. As many articles in primarily postmenopausal women, however, show no association between estrogen levels and breast density [34,35], it seems likely that local estrogen production in the breast rather than circulating estrogen levels are related to dense tissue. This also fits with the finding of Tamimi *et al. *who showed that high circulating estrogen levels and high breast density increase breast cancer risk independently from one another [36]. Evidence for local estrogen production being responsible for high breast density was found by Vachon *et al. *[37], who showed higher aromatase activity in dense than in non-dense tissue. An independent harmful effect of fatty breast tissue has not been described before, but could be explained by the fact that the fat tissue is an important source of local estrogens in the breast [24-27].

A large area of fatty breast tissue could also increase breast cancer risk through adipocytokines, such as leptin and adiponectin, which are secreted by the fat tissue. Leptin promotes breast cancer cell growth, whereas adiponectin reduces cell proliferation and enhances apoptosis. A higher body mass index is associated with increased secretion of leptin and decreased secretion of adiponectin [38]. The balance between leptin and adiponectin might also be an important factor in the development of breast cancer as described in the review by Grossmann *et al. *[39].

Currently, two studies have investigated the role of the breast fat tissue with different results. Stone *et al. *investigated the role of the non-dense area in a group of women in the United Kingdom comparable to our study population. Although the highest quintile of non-dense area gave a somewhat higher odds ratio than the other quintiles, they did not find a significant association between non-dense area and breast cancer risk. Stone *et al. *concluded that the model with the dense area alone was the most parsimonious model [19]. In this study, the results could only be adjusted for age and dense area and not for other breast cancer risk factors. When we only adjusted for age and dense area like Stone did, we observed a weaker effect than in our fully adjusted models; however, it is still statistically significant (OR 1.8, 95% CI 1.1 to 2.7 vs. 2.4, 95% CI 1.3 to 4.2). Therefore, this cannot entirely explain the discrepant results.

Stuedal *et al. *studied the role of the non-dense tissue indirectly by investigating the effect of the breast size on the relationship between mammographic density and breast cancer risk. They found that the association between breast density and breast cancer risk was weaker in women with larger breasts. This could indicate that the fat tissue is protective against breast cancer [18]. This discrepancy between their study and ours could potentially be explained by differences in study populations. In our study, the mean age of the women was 59 years old and only postmenopausal women were included. The study population of Stuedal and colleagues was considerably younger, namely 49 years old on average and most women were still premenopausal. In postmenopausal women, estrogens are mainly synthesized in the fat tissue, through conversion of androgens, whereas in premenopausal women, estrogens are mainly synthesized in the ovary [38]. This may explain why in our postmenopausal population a large area of fat tissue in the breast is related to higher breast cancer risk, while this was not observed in the study of Stuedal *et al*.

According to Stuedal *et al*., their findings might also be explained by a higher proportion of dense tissue in larger breasts having a more 'supportive' role than it does in smaller breasts and, therefore, it may be less correlated with the number of epithelial cells at risk and, hence, it is more weakly associated with breast cancer risk [18]. If true, this too may be different for pre- and postmenopausal women. It has been suggested before that high density in postmenopausal women may represent something different from high density in premenopausal women [34]. The group of premenopausal women in our study was too small to perform a separate analysis upon to further explore this explanation.

A strength of our case-control study is that it is nested in a large cohort study, in which mammograms and questionnaire information were collected long before breast cancer developed, reducing the chance of recall bias. The questionnaires contained extensive information about the potential breast cancer risk factors, allowing extensive confounder adjustment. Also, due to the long follow-up time, we were able to study breast density well before diagnosis, making it unlikely that density is influenced by the presence of a tumor, or that our findings are influenced by so-called masking bias [40]. A weakness of our older study population is the limited number of women with a very high percent density. This is inherent to the Dutch screening programme, which is restricted to women between the ages of 50 and 75 years old. Another limitation is that for this study we only have film-screen mammograms at our disposal. This has the inherent disadvantage that technical characteristics and breast thickness are not taken into account which could give less precise estimates for the dense and non-dense tissue [41]. Full field digital mammography, which has been routinely used in the Dutch screening programme for a few years, is likely to provide a more precise estimate of dense and non-dense tissue volume in the coming years.

## Conclusions

We observed that besides the size of dense tissue, the size of non-dense tissue also plays a role in the development of breast cancer. When studying dense breast tissue in relation to breast cancer risk, adjustment for non-dense tissue may give more valid results than adjustment for BMI. Although not statistically significant, the results also give some indication that a large area of fibroglandular tissue could be related to higher breast cancer risk when surrounded by a large, compared to a small area of fat tissue. Further research is warranted to confirm this effect.

## Abbreviations

BMI: Body Mass Index; EPIC: European Prospective Investigation into Cancer and Nutrition; HT: hormone therapy; IKMN: Regional Cancer Registry; IQR: interquartile range; LKR: National Cancer Registry; MLO: mediolateral oblique mammograms; OR: odds ratio; RERI: relative excess risk; SD: standard deviation.

## Competing interests

The authors declare that they have no competing interests.

## Authors' contributions

ML collected and assessed the mammograms, carried out the analysis and drafted the manuscript. PH is responsible for the initiation and follow-up of the cohort, for providing input on the interpretation of the results and helping to draft the manuscript. LP participated in discussions of the project and revised it critically for important intellectual content. GH participated in designing the study and helped edit the manuscript. WB provided input on the interpretation of the results from a radiological point of view and helped edit the manuscript. CG designed the study, provided input on the interpretation of the results and helped to draft the manuscript. All authors commented on the draft manuscript and approved the final manuscript.
